# Decamethoxin and chlorhexidine bigluconate effect on the adhesive and biofilm-forming properties of *Streptococcus mitis*

**DOI:** 10.3389/froh.2023.1268676

**Published:** 2023-11-07

**Authors:** Mariia O. Faustova, Yuliia V. Chumak, Galina A. Loban’, Maiia M. Ananieva, Viktor M. Havryliev

**Affiliations:** ^1^ Department of Microbiology, Virology and Immunology, Poltava State Medical University, Poltava, Ukraine; ^2^ Department of Surgical Dentistry and Maxillo-Facial Surgery, Poltava State Medical University, Poltava, Ukraine

**Keywords:** infectious complication, post-extraction complication, oral microbiota, antiseptics, biofilm

## Abstract

**The aim of the study:**

Was to investigate the effect of antiseptics on the adhesive and biofilm-forming properties of clinical *S.mitis* isolates isolated from the oral cavity of patients with an infectious and inflammatory post-extraction complication.

**Materials and methods:**

Twenty four clinical isolates of *S.mitis* isolated from patients were studied. The studied antiseptics included 0.02% aqueous solution of decamethoxin and 0.05% solution of chlorhexidine bigluconate. Adhesion of clinical isolates under the action of decamethoxin and chlorhexidine bigluconate was determined by the method of V.I. Brillis. The biofilm-forming properties of clinical isolates were studied using the “microtiter plate test” according to G.D. Christensen.

**Results:**

The studied clinical isolates of *S.mitis* are classified as highly adherent microorganisms. Action of decamethoxin on clinical isolates decreases the adhesion index of the studied isolates in comparison with the adhesion index of the control culture. Action of chlorhexidine bigluconate on *S.mitis* isolates increases of adhession of the studied clinical isolates in comparison with the control. After the effect of decamethoxin, the optical density of clinical isolates decreased considering the optical density results of the control. The clinical isolates left an average film-forming capacity even after chlorhexidine bigluconate action.

**Conclusions:**

Clinical isolates of *S.mitis* are highly adherent microorganisms. The antiseptic decamethoxin decreases the adhesion index of these bacteria, while chlorhexidine bigluconate increases the adhesion index of clinical *S.mitis* isolates. Clinical *S. mitis* isolates have an average biofilm formation capacity index. The antiseptic decamethoxin inhibits the biofilm formation capacity of *S.mitis* from medium to low.

## 1. Introduction

With the spread of antibiotic resistance era, the failure of antibiotic treatment of purulent inflammatory processes with bacterial flora as the etiological factor has increased. The ineffectiveness is related to the evolutionary development of microbial resistance to antibiotics (1–4). According to the literature, bacterial resistance to antibiotics can develop due to the ability of certain species of microorganisms to form biofilms. Biofilms allow bacterial cells to become protected from the effects of antibacterial drugs and also have the ability to evade the defense mechanisms of the macro-organism in individuals of different ages and sexes (5–7). A distinctive feature of bacterial infection associated with biofilm formation is not only an increased strong inflammatory response with release of significant amounts of inflammatory mediators, but also the resistance of microorganisms to antibiotics, which in turn influences the choice of these drugs for treatment of the pathological process and plays a significant role in enhancing the pathogenicity factors, namely, the adhesive properties of the pathogen (8–10). It is through bacterial adhesion that the process of microbial-macroorganism interaction begins, involving the attachment of the bacterial agent and its subsequent colonization of macroorganism cells, which in turn can be a starting mechanism for the process of biofilm formation (11, 12). Doctors are now increasingly using antiseptics to treat and prevent infections and inflammatory processes, given that microbial resistance to these drugs is acquired less frequently. In addition, some antiseptics have been shown to reduce bacterial adhesion, or even block it altogether (3, 13).

Alveolitis or dry socket is a local post-extraction complication, the etiological factor of which is the bacterial flora of the extraction site (14–18).

Currently, many scientific studies have appeared regarding the role of viridans group *streptococci,* in particular *Streptococcus mitis (S.mitis)*, in the development of infectious processes (19). These Gram-positive catalase-negative *α*-hemolytic oral streptococci belong to the opportunistic resident microbiota of the human oral cavity (20). *S.mitis* has been identified among the causative agents of various bacterial infections and their complications, such as caries, endocarditis, endophthalmitis, bacteremia, septicemia (21–24). It should be noted that *S. mitis* has the ability to make a significant contribution to the pathological process by increasing the pathogenicity of representatives of polymicrobial bacterial associations comprising biofilms. In fact, bacteria composing biofilms undergo evolutionary development together, which leads to an increase in metabolic activity and activation of virulence factors of its representatives, i.e., *S. mitis* participates in the process of co-aggregation and formation of a multispecies microbial biofilm layer (25, 26).

Today, the use of antiseptics in the treatment of inflammatory diseases of the oral cavity is promising on the background of antibiotic resistance. Chlorhexidine bigluconate is the most widely used antiseptic in dentistry recently. However, more and more often there are data on a decrease in its effectiveness. Along with this, antiseptics based on quaternary ammonium compounds (decamethoxine), possessing a wide spectrum of antibacterial action, demonstrate positive results in the prevention and treatment of infectious inflammatory processes, including those of the oral cavity (18).

The aim of the study was to investigate the effect of antiseptics on the adhesive and biofilm-forming properties of clinical *S.mitis* isolates isolated from the oral cavity of patients with an infectious and inflammatory post-extraction complication.

## 2. Materials and methods

### 2.1. Study population and culture collection

120 patients were treated for infectious and inflammatory post-extraction complications (dry socket) in the medical and surgical department of the Poltava Regional Dental Centre—Dental Clinical Polyclinic. Inclusions criteria were inflammation after tooth extraction, subject to consent to participate in the study. Exclusions criteria were other inflammation in oral cavity, diabetes, the presence of congenital or acquired immunodeficiencies, mental disorders, taking antibiotics the day before the collection material from the source of infection and refusal to participate in the study. The material was taken from the post-extraction socket on the second or third day after extraction using a tampon-probe. The stamen probe was placed in Amies transport medium for transportation to the laboratory of the Microbiology, Virology and Immunology Department of Poltava State Medical University.

The specimens were cultivated on Columbia agar with 5% sheep blood (bioMarieux, France) at 37°C under aerobic atmosphere with 10% CO_2_ for 24 h. The number of colony-forming units (CFUs) was counted using the standard plate method. The conclusion about the etiological significance of the microorganism in the infectious-inflammatory process was made after isolation of microorganism in a monoculture or in the amount of 10^6^ CFUs or more, followed by its species identification. The final identification was performed by using a Vitec-2 compact bioMarieux automatic bacteriological analyzer (France) according to the manufacturer's instructions. Twenty four clinical isolates of *S.mitis* isolated from patients were the object of the study; 5 strains of *S. mitis* were isolated from the oral cavity of somatically healthy individuals as a control for determining the biofilm-forming and adhesive properties of the studied isolates.

At the beginning of the study, each patient signed an informed consent regarding the collection of material for the study, the steps of the study and the possible consequences. The study was performed in accordance with the Helsinki Declaration on Ethical Principles of Medical Research Involving Human Subjects and approved by the Commission on Biomedical Ethics of Poltava State Medical University (Minutes No. 188 of 25.11.2020).

### 2.2. Studied antiseptics

The studied antiseptics included 0.02% aqueous solution of decamethoxin (Decasan produced by Yuria-Pharm Ukraine Ltd.); 0.05% solution of chlorhexidine bigluconate (Chlorhexidine produced by Pharmaceutical Factory Vishpha Ukraine Ltd.). In the study, we used average values of sub-bacteriostatic concentrations of the above antiseptics for *S.mitis*, which were taken as ¼ of their minimum inhibitory concentrations for the studied microorganisms and determined by serial dilution method according to the recommendations of ISO standard 20776-1:2019. The sub-bacteriostatic concentration for decamethoxin was 0.23 ± 0.3 µg/ml, the sub-bacteriostatic concentration for chlorhexidine bigluconate was 0.90 ± 0.6 µg/ml.

### 2.3. Determination of anticeptics effect on the adhesive properties of microorganisms

Adhesion of clinical isolates under the action of decamethoxin and chlorhexidine bigluconate was determined by the method of V.I. Brillis using blood group I (0) Rh + erythrocytes (27).

One drop of the overnight bacterial culture was suspended in one drop of buffer solution, followed by the introduction of one drop of erythrocyte suspension (concentration 100 million/ml) on a glass slide. The slide was placed in a humid chamber at 37°C for 30 min, dried, fixed by ethanol for 10 min. and stained by Gram. Adhesive properties were assessed using the microbial adhesiveness index (MIA) by counting the average number of microbial cells attached to one erythrocyte involved in the adhesive process. The number of microbial cells attached to one erythrocyte was counted, evaluating 50 erythrocytes, with no more than 5 in one field of view using a microscope MOCROmed XS—3330 (100X/1.25).

According to Brillis's method, all bacteria were subdivided, according to the adhesion index criterion, into microorganisms without adhesion (with an adhesion index of ≤1.75), those with low adhesion (with an adhesion index of 1.75–2.49), medium adhesion (with an adhesion index of 2.50–4.0), and those with high adhesion (with an adhesion index >4.0) (25). The effect of sub-bacteriostatic concentrations of decamethoxin and chlorhexidine bigluconate on the adhesive properties of the clinical isolates studied was examined by comparing changes in MIA in the presence of antiseptics compared with MIA without them. 5 strains of *S. mitis* isolated from the oral cavity of somatically healthy individuals were used as a control for determining the adhesive activity of the studied isolates. Studies with control strains of *S.mitis* were repeated three times.

### 2.4. Determination of anticeptics effect on the biofilm-forming properties of microorganisms

The biofilm-forming properties of clinical *S. mitis* isolates were studied using the MtP-test “microtiter plate test” according to G.D. Christensen (28). Biofilms were reproduced in wells of sterile flat-bottomed 96-well polysterol plate (Corning, USA) and stained with 1% crystal violet solution. The ability of microorganisms to form biofilms was assessed by dye absorption in density units using a microplate photometer (Labline-026, Austria) at 630 nm. The ability of microorganisms to form biofilms was evaluated using the following criteria: low (optical density <0.120), medium (optical density = 0.121- 0.239) and high (optical density >0.240) (28). 5 strains of *S. mitis* isolated from the oral cavity of somatically healthy individuals were used as a control for determining the biofilm-forming properties of the studied isolates. Studies with control strains of *S.mitis* were repeated three times.

To assess the effect of decamethoxin and chlorhexidine bigluconate on the film-forming ability of *S.mitis*, sub-bacteriostatic concentrations of the preparations were added to the bacterial culture immediately after introduction to the wells of the plate. The optical density of the test isolates without antiseptics was used as a control against which the film-forming results of clinical isolates in the presence of antiseptics were compared.

Studies with antiseptics on the adhesive and film-forming properties of *S.mitis* were repeated three times.

### 2.5. Statistical analysis

Variation-statistical processing of the results of the study was performed using Microsoft Excel 2019 with determination of the main variation indicators: mean values (M), mean errors (m), *p*-value (p). Student's t-test was used to compare two normally distributed groups. Univariate variance analysis (ANOVA: single factor) was used to compare the results of three or more groups of data. The Bonferroni correction adjusted a significance level to control the overall probability of errors (false positive) for multiple hypothesis tests. The result was considered significant reliably if the *p*-value was less than 0.05.

## 3. Results

We found that the studied clinical isolates of *S.mitis* isolated from the oral cavity of patients with infectious and inflammatory post-extraction complications are classified as highly adherent microorganisms (Table 1). The MIA for the studied isolates was 8.55 ± 0.82. Moreover, isolates of *S. mitis* that colonized the oral cavity of somatically healthy adults and were used as a control also showed a high ability to adhere. The average MIA for them was 8.16 ± 0.45, and the results had no significant difference compared to the results of MIA of the studied isolates.

**Table 1:** Microbial adhesion index (MIA) of clinical strains of *S.mitis* isolated from patients with dry socket and control isolates of healthy adults (M ± m).

Control isolates			Tested isolates		
No	Isolate	MIA	No	Isolate	MIA
1	*S.mitis* C1	7.9	1	*S.mitis* 01	9.7
2	*S.mitis* C2	8.8	2	*S.mitis* 02	7.9
3	*S.mitis* C3	7.6	3	*S.mitis* 03	7.7
4	*S.mitis* C4	8.5	4	*S.mitis* 04	8.9
5	*S.mitis* C5	8	5	*S.mitis* 05	9.7
6	*S.mitis* C6	7.9	6	*S.mitis* 06	7.9
7	*S.mitis* C7	8.8	7	*S.mitis* 07	7.7
8	*S.mitis* C8	7.6	8	*S.mitis* 08	8.9
9	*S.mitis* C9	8.5	9	*S.mitis* 09	9.7
10	*S.mitis* C10	8.0	10	*S.mitis* 10	7.9
11	*S.mitis* C11	7.9	11	*S.mitis* 11	7.7
12	*S.mitis* C12	8.8	12	*S.mitis* 12	8.9
13	*S.mitis* C13	7.6	13	*S.mitis* 13	9.7
14	*S.mitis* C14	8.5	14	*S.mitis* 14	7.9
15	*S.mitis* C15	8.0	15	*S.mitis* 15	7.7
			16	*S.mitis* 16	8.9
			17	*S.mitis* 17	9.7
			18	*S.mitis* 18	7.9
			19	*S.mitis* 19	7.7
			20	*S.mitis* 20	8.9
			21	*S.mitis* 21	9.7
			22	*S.mitis* 22	7.9
			23	*S.mitis* 23	7.7
			24	*S.mitis* 24	8.9
Average		8.16 ± 0.45	Average		8.55 ± 0.82

After action of sub-bacteriostatic concentration of decamethoxin on clinical isolates, a 1.3-times (*p* < 0.05) decrease of the MIA of the studied isolates was observed in comparison with the adhesion index of the free-antiseptic culture. Taking into account the results of action of chlorhexidine bigluconate sub-bacteriostatic concentration on *S.mitis* isolates, we revealed 1.2-times (*p* < 0.05) increase of MIA parameters of the studied clinical isolates in comparison with MIA parameters of the *S.mitis* with no antiseptics. When comparing MIA parameters in the presence of sub-bacteriostatic concentrations of decamethoxin and chlorhexidine bigluconate between each other, it was found that MIA parameters after the action of sub-bacteriostatic decamethoxin concentration were 1.5 times (*p* < 0.05) lower than MIA parameters after sub-bacteriostatic concentration of chlorhexidine bigluconate (Table 2).

**Table 2:** Microbial adhesion index of clinical *S.mitis* isolates under sub-bacteriostatic concentrations of the antiseptics tested (M ± m).

Isolates	No antiseptics	Decamethoxin	Chlorhexidine bigluconate
*S.mitis* 01	9.7	7.5	11.6
*S.mitis* 02	7.9	5.6	8.6
*S.mitis* 03	7.7	5.7	11.2
*S.mitis* 04	8.9	5.4	10.2
*S.mitis* 05	9.7	8.6	11.6
*S.mitis* 06	7.9	7.5	8.6
*S.mitis* 07	7.7	6	10
*S.mitis* 08	8.9	6	11.2
*S.mitis* 09	9.7	7.5	11.6
*S.mitis* 10	7.9	5.6	8.6
*S.mitis* 11	7.7	5.7	11.2
*S.mitis* 12	8.9	5.4	10.2
*S.mitis* 13	9.7	8.6	11.6
*S.mitis* 14	7.9	7.5	8.6
*S.mitis* 15	7.7	6	10
*S.mitis* 16	8.9	6	11.2
*S.mitis* 17	9.7	7.5	11.6
*S.mitis* 18	7.9	5.6	8.6
*S.mitis* 19	7.7	5.7	11.2
*S.mitis* 20	8.9	5.4	10.2
*S.mitis* 21	9.7	8.6	11.6
*S.mitis* 22	7.9	7.5	8.6
*S.mitis* 23	7.7	6	10
*S.mitis* 24	8.9	6	11.2
Average	8.55 ± 0.82	6.53 ± 1.11*^,^^#^	10.37 ± 1.18*

* Reliability of microbial activity index with antiseptic control and microbial activity index with control (*p* < 0.05).

^#^ Sufficiency of different culture adhesion index and decamethoxin presence with culture adhesion index and chlorhexidine bigluconate presence (*p* < 0.05).

**Figure 1:**
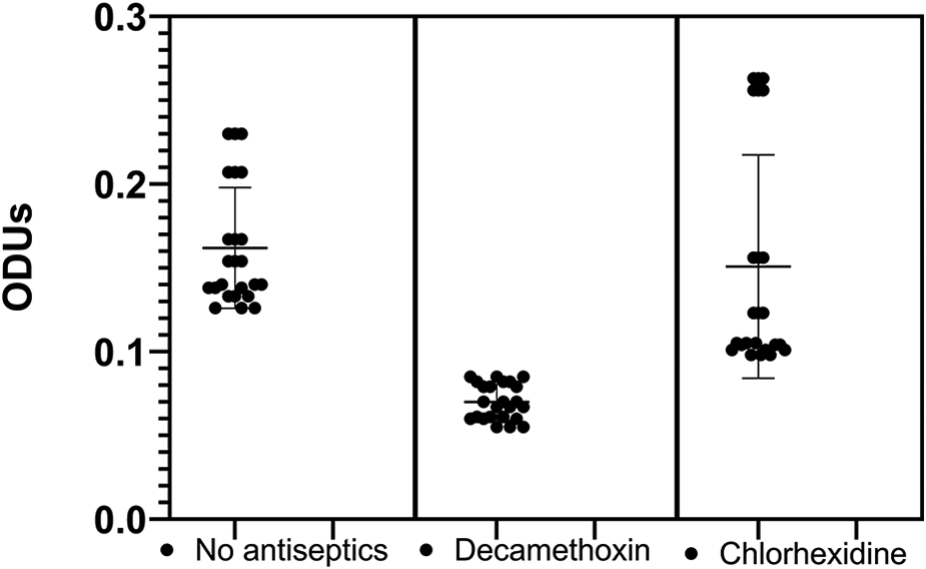
Optical density values of clinical *S.mitis* isolates at sub-bacteriostatic concentrations of the antiseptics tested (M ± m).

The next stage of our study was to determine the effect of sub-bacteriostatic concentrations of the antiseptics decamethoxin and chlorhexidine bigluconate on the biofilm-forming properties of clinical *S. mitis* isolates. Having evaluated the results of the study it can be stated that the studied clinical isolates of *S. mitis* according to the methodology of G.D. Christensen referred to microorganisms with medium ability to biofilm formation (Table 3). It should be noted that, on average, the parameters of biofilm-forming properties of the studied isolates did not differ significantly from the given parameters of control isolates from healthy individuals. After the effect of a sub-bacteriostatic concentration of decamethoxin, we found that the optical density of clinical isolates decreased 2.3-times (*p* < 0.05) considering the optical density results of the *S.mitis* with no antiseptics, i.e., the biofilm formation capacity of the studied isolates became low (Figure 1).

**Table 3:** Optical density values of clinical strains of *S.mitis* isolated from patients with dry socket and control isolates of healthy adults (M ± m).

Control isolates			Tested isolates		
No	Isolate	ODUs	No	Isolate	ODUs
1	*S.mitis* C1	0.147	1	*S.mitis* 01	0.167
2	*S.mitis* C2	0.163	2	*S.mitis* 02	0.23
3	*S.mitis* C3	0.166	3	*S.mitis* 03	0.138
4	*S.mitis* C4	0.185	4	*S.mitis* 04	0.154
5	*S.mitis* C5	0.141	5	*S.mitis* 05	0.126
6	*S.mitis* C6	0.147	6	*S.mitis* 06	0.140
7	*S.mitis* C7	0.163	7	*S.mitis* 07	0.133
8	*S.mitis* C8	0.166	8	*S.mitis* 08	0.207
9	*S.mitis* C9	0.185	9	*S.mitis* 09	0.167
10	*S.mitis* C10	0.141	10	*S.mitis* 10	0.230
11	*S.mitis* C11	0.147	11	*S.mitis* 11	0.138
12	*S.mitis* C12	0.163	12	*S.mitis* 12	0.154
13	*S.mitis* C13	0.166	13	*S.mitis* 13	0.126
14	*S.mitis* C14	0.185	14	*S.mitis* 14	0.140
15	*S.mitis* C15	0.141	15	*S.mitis* 15	0.133
			16	*S.mitis* 16	0.207
			17	*S.mitis* 17	0.167
			18	*S.mitis* 18	0.230
			19	*S.mitis* 19	0.138
			20	*S.mitis* 20	0.154
			21	*S.mitis* 21	0.126
			22	*S.mitis* 22	0.140
			23	*S.mitis* 23	0.133
			24	*S.mitis* 24	0.207
Average		0.160 ± 0.02	Average		0.162 ± 0.04

Our study showed no significant effect of chlorhexidine bigluconate on the biofilm formation of the examined microorganisms compared to *S.mitis* with no antiseptics. The clinical isolates left an average film-forming capacity even after antiseptic action. By evaluating the effect of sub-bacteriostatic concentrations of the studied antiseptics on the change in optical density, we determined that after the action of sub-bacteriostatic concentration of decamethoxin, the optical density decreased 2.1-times (*p* < 0.05), compared with the action of sub-bacteriostatic concentration of chlorine. Such results indicate that decamethoxin changes the biofilm formation capacity of clinical isolates from medium to low.

## 4. Discussion

Microbial adhesion starts a process of interaction between the microorganism and the macroorganism, and such contact can cause a pathological process. Sarah E. Whitmore notes in her research that *S. mitis* possesses a variety of properties that contribute to different forms of co-existence with other microorganisms. The author attributes this existence to the adhesive properties of this group of bacteria (25). In our studies, clinical isolates of *S. mitis* isolated from patients with infectious and inflammatory post-extraction complications (dry socket) were classified as highly adhesive microorganisms according to the adhesion index by Brillis. In addition, our study found that the antiseptic decamethoxin under study has the ability to reduce the adhesive properties of clinical *S.mitis* isolates, while the antiseptic chlorhexidine bigluconate not only did not reduce the adhesion of *S.mitis* isolates, but also even increased it. Such data should be considered in clinical practice when choosing an antiseptic for the treatment and prevention of infectious and inflammatory processes. Ruben Barreto points out in his research that micro-organisms have the ability to acquire resistance to antiseptics, even those of widespread use. Chlorhexidine bigluconate was added to the list of antiseptics investigated and resistance to it was also found by microorganisms (29). It is possible that resistance to bigluconate chlorhexidine is responsible for various mechanisms leading to an increase in the adhesive properties of bacteria. Oleksandr Nazarchuk et al. studied the film-forming ability of Gram-negative bacteria under the action of the antiseptic decamethoxin in their research (30). Their data showed that decamethoxin reduced the biofilm formation ability of the Gram-negative microorganisms studied. At the same time, given the results of our study, the antiseptic decamethoxin was found to have the ability to reduce biofilm formation of Gram-positive bacteria as well. These findings should be considered in the treatment and prevention of infectious and inflammatory processes in which *S. mitis* is involved. After all, it is the biofilm that protects micro-organisms from the effects of antibacterial drugs, which entails further development of the pathological process, as well as increasing the resistance of bacteria to antibiotics in it. And using the antiseptic decamethoxin can reduce the biofilm-forming properties of *S.mitis* if this group of streptococci is one of the representatives of the polymicrobial biofilm community of the post-extraction dental extraction site.

## 5. Conclusion

Thus, clinical isolates of *S.mitis* isolated from patients treated for infectious inflammatory post-extraction complications (dry socket) are highly adherent microorganisms. The antiseptic decamethoxin decreases the adhesion index of these bacteria, while chlorhexidine bigluconate conversely increases the adhesion index of clinical *S.mitis* isolates. Clinical *S. mitis* isolates have an average biofilm formation capacity index. The antiseptic decamethoxin inhibits the biofilm formation capacity of *S.mitis* from medium to low.

## Data Availability

The raw data supporting the conclusions of this article will be made available by the authors, without undue reservation.
